# Developing a Competent Workforce for Integrated Health and Social Care: What Does It Take?

**DOI:** 10.5334/ijic.2533

**Published:** 2016-10-28

**Authors:** K Viktoria Stein

**Affiliations:** International Foundation for Integrated Care, 7200 The Quorum, Oxford Business Park North, Garsington Road, Oxford OX4 2JZ, GB

## Summary

Reflecting on the knowledge, skills and attitudes necessary to work in integrated care, this perspectives paper explores the competencies required to implement and deliver integrated care and analyses how current education and training approaches fall short of conveying these competencies on all levels. By defining the differences between knowledge, skills and attitudes, and outlining the key ingredients for a competent workforce, this paper brings to light one of the most neglected topics in integrated care.

## Why is building a competent workforce not a priority yet?

Nobody would ever question the necessity of having a competent workforce to deliver high quality services. Equally, everyone keeps reaffirming the challenges and complexities associated with designing, managing, implementing and delivering integrated care. Yet, there are few examples of integrated care initiatives, which invest in the education and training of their people or which have adequate programmes available to build the skills and attitudes necessary to change service delivery towards integrated, people-centred care. While the focus remains on implementing a plethora of tools and instruments to support and foster integration of health and social services, little thought is given to the people who need to implement and utilize these tools on a day-to-day basis.

Having started my professional career as a PhD and PostDoc at the Medical University of Vienna, I have been actively involved in the education and training of students and professionals from the outset. I have designed and delivered courses from the undergraduate to the professional training level, and for uni- as well as multi-disciplinary audiences around the world. And as a health economist with a background in organisational development and project management, I have always been fascinated by how our health and social systems are organised with a seeming disregard for an overall understanding and alignment of activities. Combining my passion for teaching with my long-standing involvement in integrated care, it is my sincere conviction that we will not be able to establish sustainable integrated care solutions, if we do not change the way and the philosophy behind education and training of our professionals.

## What are competencies and how do we acquire them?

Based on [1] eponymous paper, with which he founded the movement for competency-based education, the “Iceberg model” very aptly visualises the complexity of competencies by distinguishing between technical competencies, what we know and can do (i.e. the iceberg we see above the surface), and behavioural competencies, what we perceive and what motivates us (i.e. the iceberg below the surface). The former comprise knowledge and skills and can be directly influenced by education and training; the latter are our attitudes, which can only be influenced indirectly through education, training and role models. In a paper for the [2] describe health workforce competencies as “…essential complex knowledge based acts that combine and mobilize knowledge, skills, and attitudes with the existing and available resources to ensure safe and quality outcomes for patients and populations. Competencies require a certain level of social and emotional intelligence that are as much flexible as they are habitual and judicious.” The authors continue by listing key features of competencies, including that they take time to acquire, they must be measurable and flexible, and they are a distinguishing feature for groups – which underlie the emergence of professional and/or organisational cultures (see also [3] in this issue).

## Workforce competencies for integrated care

In a literature review [4], summarised the workforce changes taking place during the implementation of integrated care models. These included new leadership and management roles (as in nurse-led care or case management), new professional roles (as in pharmacist involvement), and new working environments (as in inter-disciplinary team meetings or multi-disciplinary pathways). So, not surprisingly, integrated care very often challenges the way professionals have been taught to deliver care as roles and responsibilities are redefined, new tools and processes implemented, and cross-professional and cross-sectoral collaboration formalised. This may lead to resistance, resignation or disregard [5]. In order to enable professionals to fill these new roles, to manage health and care rather than disease and cure, or to work in teams across professions and sectors, they need to acquire a different set of knowledge, skills and attitudes from what they have traditionally been trained to do. Numerous authors have hence highlighted that education and training is a key principle of integrated care (see for example [6,7,8,9,10]). By supporting and training staff to work in an inter-disciplinary and integrated environment, a gradual change of organisational and professional cultures may also be achieved, thus enabling long-term transformation of service delivery and creating a common understanding and culture conducive to integrated care [11,3].

## Building competencies needs a holistic approach

Developing education and training programmes to build a competent workforce for integrated care needs a multi-faceted approach on all levels. On the system and organisational levels, policy makers and managers need to create a framework for learning environments and organisations, in which providers can work together and engage with patients, caregivers and communities. This importantly entails creating trust and having respect for one another, as well as developing an open error management to support the learning organisation. But it also means sharing responsibilities and committing oneself to the active involvement in a continuous learning cycle [2].

It is also paramount that all professionals on all levels need to participate in this competency-based learning. Currently, if there is any emphasis laid, it is still on the health workforce [2,12], but it is clear that everyone involved will need to acquire additional competencies, if transformational change for integrated care is to be achieved. This encompasses decision makers and leaders embracing a common vision for integrated care and providing an adequate regulatory framework, more professional bodies and associations to engage in the development of competency frameworks for education (see for example the work of the Committee of Social Workers Education [13]), and people and communities actively taking part in the design and delivery of integrated care (see [14] in this issue). And so indeed this quote from [12] may be adapted to read that “…all (…) professionals in all countries [need] to be educated to mobilize knowledge and to engage in critical reasoning and ethical conduct so they are competent to participate in patient and population-centred health systems as members of locally responsive and globally connected teams.”

## The way forward – changing competencies to change the system

A key experience for me was when I was teaching 5^th^ year medical students public health and health systems organisation. One lesson was about patient empowerment and how to achieve it, when one of the students criticised the whole notion and said he would never ask a patient for their opinion because he was the one who had studied for six years. Another student added that patients wouldn’t listen anyway to what they were told, they never gave up smoking or lost weight, so what was the point in including them in the decision making. After I recovered from my astonishment, I challenged them whether any one of them had ever asked their patients WHY they hadn’t changed, or whether they had any problems in their lives, which may make it difficult to stick to the plan. Stunned silence – they had been taught all the organ groups and the corresponding diseases, they knew all the bones, sinews and muscles in the body, and they had had internships and patient contact. But it had never occurred to them (and no one had ever told them) to ask a patient the simple question of “How can I help you? How can we achieve this together?”

The students we teach today are the professionals who we expect to deliver integrated care in the future. If we don’t teach them the skills and attitudes necessary from the very beginning, we must not be surprised if integrated care so often fails. It is therefore paramount to not only introduce integrated care into the curricula of professional education, as has been done in Belgium and Serbia [15]. We also need to make inter-professional education and training the norm, not the exception, as has already been done in Canada (see for example [16,17]). At the same time, we need to ensure that professionals enter into working environments, which meet their expectations of inter-disciplinary teamwork, mutual respect and trust. Finally, we need to design continuous learning programmes geared to the enhancement of the knowledge, skills and attitudes necessary for integrated care to work on all levels. Working in health and social care is very much about (com)passion – instead of stifling that with evermore complex processes and protocols, we should foster this passion by refocusing on what it is all about: to work together for better health and wellbeing of our populations. In order to achieve this, we need competent people, who enliven the principles of integrated care.
